# On the statistical foundation of a recent single molecule FRET benchmark

**DOI:** 10.1038/s41467-024-47733-3

**Published:** 2024-04-30

**Authors:** Ayush Saurabh, Lance W. Q. Xu, Steve Pressé

**Affiliations:** 1 Center for Biological Physics, Arizona State University, Tempe, AZ USA; 2 Department of Physics, Arizona State University, Tempe, AZ USA; 3 School of Molecular Sciences, Arizona State University, Tempe, AZ USA

**Keywords:** Biological physics, Computational biophysics, Fluorescence resonance energy transfer

**arising from** M. Götz et al. *Nature Communications* 10.1038/s41467-022-33023-3 (2022)

A benchmark recently published in Nature Communications entitled “A blind benchmark of analysis tools to infer kinetic rate constants from single-molecule Förster Resonance Energy Transfer (FRET) trajectories” by ref. ^1^ compared multiple FRET analysis tools. To benchmark these tools, the organizers provided FRET tool developers synthetic training datasets, along with the data-generating Matlab script. The teams applied their own tools to the challenge datasets provided. The organizers then compared the output (such as learned kinetic parameters from the supplied data) for all tools using metrics including coefficients of variation. The tools were also applied to experimental data.

Here we demonstrate that the benchmark favors FRET tools making similar assumptions to those present in the simplified data generation process (i.e., Gaussian models analyze Gaussian noise data well) and, by the same token, leads to bias and incorrect uncertainty in parameter estimates obtained by tools incorporating physical features of FRET that are otherwise not incorporated into the data generated used to test the performance of FRET analysis tools.

As an example, realistically generated data used to test the performance of FRET analysis tools should incorporate common FRET noise sources of physical origin in order to properly benchmark tools and their robustness across signal-to-noise ratio (SNR) regimes. These sources include, as examples, photon shot noise, detector noise, and spectral crosstalk^2^ just to name a few. Instead, the data generated by the benchmark organizers lacked these features and were instead generated under Gaussian noise assumptions. Here we investigate the consequences of evaluating the performance of FRET analysis tools on data excluding realistic FRET features by testing on participating tools that performed well in most of the benchmark tests, Hidden-Markury^3^ and MASH-FRET^4,5^, on data we simulate with just two realistic and widespread features: Poisson shot noise and detector crosstalk.

In doing so, we demonstrate that: (1) benchmark participants, relying on a Gaussian noise model, predictably do well when tested on Gaussian noise data like the data generated for the benchmark (Fig. 1) and, as a consequence, adding physical features to one’s analysis, such as Poissonian noise, results in inaccurate parameter and uncertainty estimates as it creates a mismatch between the model used to analyze the data and the data analyzed; (2) conversely, benchmark participants making Gaussian noise assumptions, are outperformed by standard Hidden Markov models (HMMs) equipped with a correct physical Poisson noise model when Poisson shot noise is present in the data (Fig. 1); and (3) the benchmark participants exhibit important biases in the presence of spectral crosstalk, one of many widespread FRET features left untested in the benchmark (Fig. 2).

**Fig. 1:**
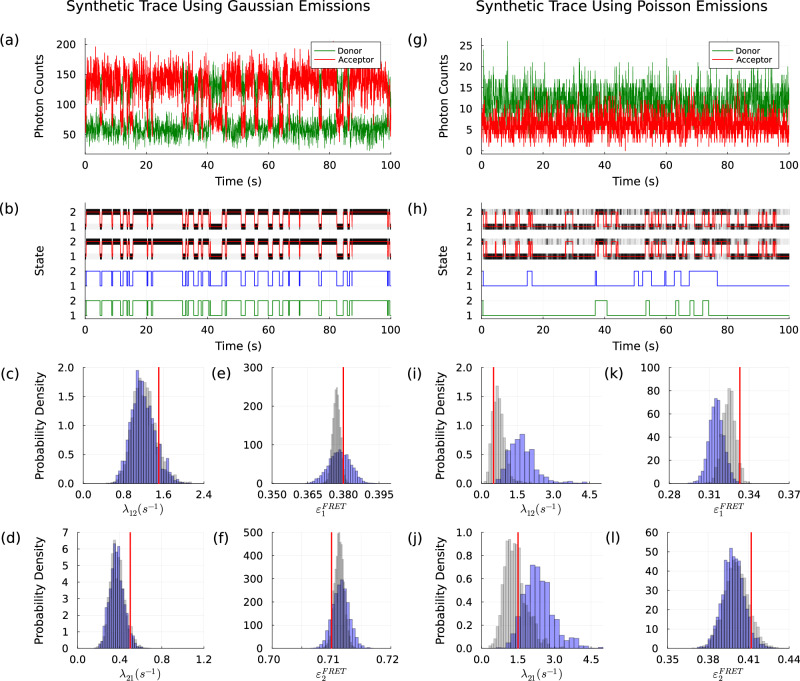
Comparison of analysis strategies for estimating kinetic rates (*λ*_12_ and *λ*_21_) and FRET efficiencies (${\varepsilon }_{1}^{FRET}$ and ${\varepsilon }_{2}^{FRET}$) for a two-state system. **a** A high-SNR synthetic FRET trace generated for a two-state system with FRET efficiencies 0.38 and 0.71 using Gaussian emission model; Kinetic rates *λ*_12_ = 1.5 s^−1^ and *λ*_21_ = 0.5 s^−1^; Gaussian means (per bin) of ~125 and 58 for the donor channel with standard deviations of 21 and 13. Acceptor channels means are set to 77 and 142, with standard deviations of 15 and 21, respectively. We set the bin size to 50 ms. **b** Ground truth trajectory in red, grayscale bands show probability (higher for darker color) for the system to be in either of the two states. The bands at the top show probabilities estimates using a Gaussian emission HMM. The bands immediately below show probabilities estimated using Poisson emission HMM. The two trajectories at the bottom are estimated by MASH-FRET^4,5^ (blue) and Hidden-Markury^3^ (green). The blue and gray histograms (**c**–**f**) for kinetic rates and FRET efficiencies correspond to the HMMs equipped with Gaussian and Poisson emission models, respectively. Ground truth in red. **g** A low-SNR synthetic FRET trace generated using Poisson distributions with means of 10 and 8 for the donor channel, and 12 and 5 for the acceptor channel. FRET efficiencies are 0.33 and 0.41. Same kinetic rates as for the high-SNR case. **h**–**l** show estimated trajectories and histograms for the low-SNR FRET trace in the same manner as panels (**b**–**f**).

**Fig. 2:**
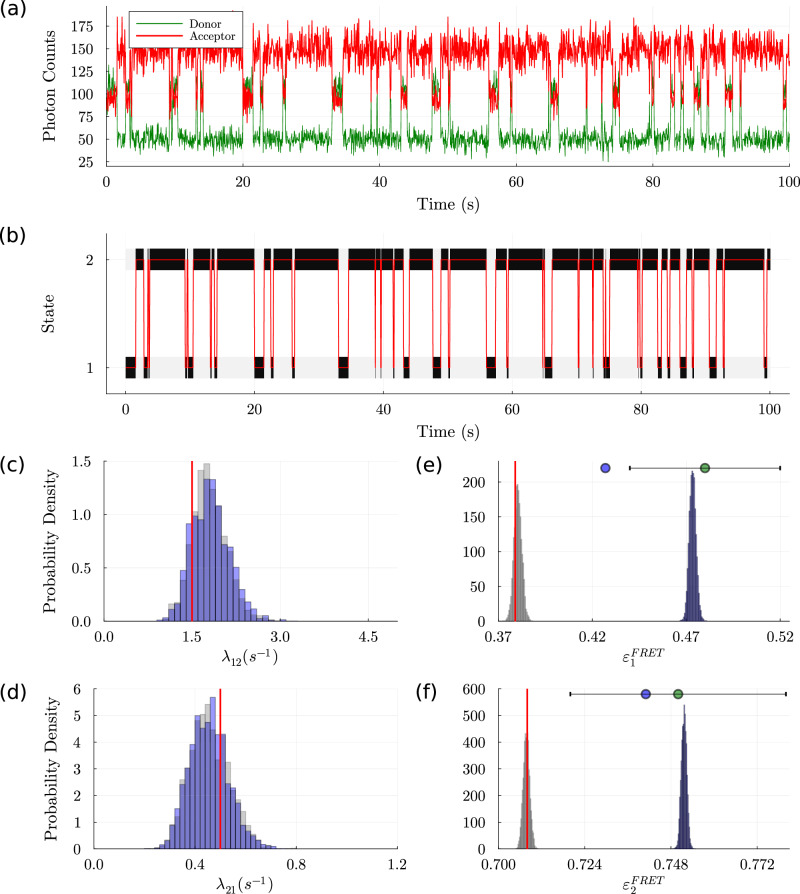
Comparison of analysis strategies from FRET trace with spectral crosstalk for a two-state system. The top panel FRET trace (**a**) is generated with Poisson emissions for both donor and acceptor channels but with photon counts per bin for donor and acceptor approximately 124 and 58, and 75 and 141, respectively. Kinetic rates and the bin size are same as Fig. 1. All conventions (grayscale in (**b**), red lines for ground truth) are the same as Fig. 1. All methods considered (HMMs with and without crosstalk correction and Hidden-Markury as well as MASH-FRET) predicted equivalently good state trajectories as shown in (**b**) due to the low noise level. For convenience, we show the bands picked out by the Poisson emission HMM only. Kinetic parameters are shown in (**c**–**f**). HMMs using Poisson emission but otherwise assuming no crosstalk (blue histograms in **c**–**f**) predictably overestimate FRET efficiencies. Similar overestimates are recovered in Hidden-Markury that ignores crosstalk and provides error bars around the incorrect efficiency estimates shown by green circles (**e**, **f**). In MASH-FRET (blue circles, **e**, **f**), crosstalk is incorrectly modeled; more details in text. The standard Poisson emission HMM with crosstalk correction (gray histogram, **e**, **f**) outperforms the two benchmark participants.

In summary, the point here is not to question the generally broad applicability of the Gaussian assumption in FRET modeling. Rather the statistical point demonstrated through examples is that the benchmark is set up in such a way that tools whose assumptions are directly aligned with the simplified data generation process provide best parameter estimates and, by the same token, tools incorporating physical features beyond the Gaussian noise model result in inaccuracies.

## Data Simulation—Poisson shot noise

We first explore the consequences of Götz et al. using a simplified Gaussian noise model (also termed Gaussian “emission” in machine learning jargon) in the data generation process. We do so in order to demonstrate an example of how extending an analysis tool to include physical features beyond the Gaussian noise model assumed becomes problematic to this benchmark.

A Gaussian noise model is a simplifying assumption for many reasons. For example, when photon budgets are lower, photon shot noise manifests as Poissonian. Only in the limit of longer camera exposures (or larger bins in the case of binned single photon counts) is a Gaussian approximation warranted. Other reasons for non-Gaussian noise also exist. For example, detector readout given photon counts is not necessarily Gaussian, e.g., EMCCD cameras where stochasticity is introduced in two stages^6^–the EM gain, requiring a Gamma emission model and readout noise often modeled as Gaussian. Convolving these distributions leads to a final noise model often inadequately approximated by a Gaussian.

To verify how sensitive the parameter estimates of Götz et al.’s benchmark participants are to the favorable Gaussian noise conditions under which the data were generated, and conversely how physically inspired models may result in less accurate parameter estimates, we first generated synthetic FRET traces using standard Gillespie algorithm^7^, similar to the benchmark organizers, in combination with Gaussian and Poisson emission models. For simplicity only, we did not convolute the emission with additional detector noise. We considered two FRET states. For the Gaussian emission model, we considered, as a sanity check, a high-SNR case where the Poisson distribution is well approximated by a Gaussian distribution (Poisson mean >30) and all analysis tools are expected to perform well. Therefore, we selected photon count means (per bin) of approximately 125 and 58 for the donor channel with standard deviations of 21 and 13, respectively, while for the acceptor channel we considered means of approximately 77 and 142 with standard deviations of 15 and 21, respectively. For the data generated using Poisson emission, we considered donor means for both states of 10 and 8, respectively. Similarly, for both states in the acceptor channel, we chose Poisson means of 12 and 5.

We then analyzed the data in four different ways: we applied standard HMMs following textbook protocols^8,9^ under two emission models (Poisson and Gaussian). We also analyzed the data using two well-performing benchmark participants.

The results of Fig. 1 immediately confirm, as a sanity check that both Gaussian and Poisson emission HMMs as well as both Hidden-Markury^3^ and MASH-FRET^4,5^ do well for the dataset generated according to the high-SNR Gaussian noise model. More interestingly, Fig. 1 also highlights the failure of the Gaussian emission HMM in analyzing data generated with Poisson emission: indeed the Gaussian emission HMM overfits the data (by over-interpreting incorrectly modeled noise as state transitions). Figure 1 also underscores how a standard HMM equipped with a Poisson emission model outperforms the two benchmark participants (Hidden-Markury^3^ and MASH-FRET^4,5^) emphasizing the sensitivity of these FRET tools to violations of their underlying Gaussian noise assumption.

In contrast to how the HMM with a Gaussian emission model applied to the Poisson emission data overfits the data, the two benchmark participants significantly underfit transition kinetics by approximately a factor of 10 (as shown in the two bottom trajectories of Fig. 1h). This underfitting is the result of additional approximations made in these tools beyond the standard HMM (for instance, the modular structure of these FRET tools which approximates the exact global optimization as would be performed with HMMs over rate and efficiency parameters).

## Data Simulation—Spectral Crosstalk

Spectral crosstalk is another critical example of a physical feature often incorporated in quantitative FRET analyses^10^ and corrects for photon misidentification. Ignoring crosstalk in analysis tools leads to FRET efficiency estimate biases confounding quantitative FRET pair distance assessments^11^.

Here we generated synthetic data assuming 15% crosstalk from the donor to acceptor channels (with percentages motivated from ref. ^10^). We then analyzed the data incorporating and ignoring crosstalk following ref. ^11^, and the two benchmark participants used earlier; see Fig. 2. We intentionally consider high-SNR data to isolate the complicating effects of crosstalk in the subsequent analysis.

Figure 2 immediately reveals that Poisson emission HMMs with crosstalk correction outperform the benchmark participants that overestimate FRET efficiency by the ~15% predictably introduced by crosstalk. Put differently, FRET pair distances inferred using the two benchmark participants are biased. While not shown, in this high-SNR case, Gaussian emission HMMs would perform very similarly to Poisson emission HMMs (that is, successfully in the case of crosstalk correction and showing biases in the absence of correction).

Of greater concern is MASH-FRET (blue circles, e, f) where the authors attempt to correct donor to acceptor crosstalk by subtracting the acceptor intensity coinciding with donor photons detected as acceptor photons. However, these photons are not correctly added back as part of donor intensity. This is clear from their code (v1.3.3.1, the latest version at the time of submission) on line 38 of the file “crossCorr.m”^12^ as well as their online manual^13^. Therefore, while Hidden-Markury ignores crosstalk altogether, MASH-FRET still does not correct for crosstalk.

## Discussion

The point of Figs. 1 and 2 are not to say that all tools used in the benchmark should perform well in all circumstances. Then why bother with a benchmark? The organizers are not in control of the quality of the participating tools. Rather, the point we make is that it would be of the interest to the FRET community to design a benchmark capable of adequately testing and identifying tools that model features of FRET experiments to varying degrees of accuracy. In other words, an ideal benchmark should not favor tools whose assumptions coincide with those inherent in test datasets. As a direct consequence, FRET tool performances should be re-assessed.

At this stage, we should also re-visit which model assumptions, or critical coding bugs left untested, were responsible for the discrepancies between tools in the analysis of the experimental binding-unbinding data provided (Fig. 5 and Supplementary Figs. 4–6 of ref. ^1^). Equally interesting would be to re-visit the outcome of other tests in the benchmark (such as state determination not touched upon here) or the validity of conclusions drawn from experimental data using benchmark participants.

A final, more subtle, point is also in order if such a benchmark were repeated. For example, how should we compare tools producing error bars encompassing ground truth values (such as in panels (c–l) of Fig. 1) with tools reporting point parameter estimates invariably differing from the exact ground truth? Quantitative data analysis requires uncertainty around parameter estimates (for example, through Cramer-Rao lower bounds^14^ if only to accommodate finite data effects on parameter estimation).

## Methods

### Data simulation

To generate simulated FRET datasets, we first selected kinetic rates for state transitions. Then a state trajectory, defined by sequence of states and their dwell times, was generated using the standard Gillespie^7^ algorithm. Next, we discretized this state trajectory into evenly spaced time bins according to a chosen camera exposure period. The donor and acceptor channel photon emissions in each bin were then sampled from Poisson distributions with state dependent means for each channel. In the case where donor to acceptor channel crosstalk was present, the mean acceptor count was increased by adding a term multiplying crosstalk probability with mean donor emission. The mean donor count was reduced by the same amount. Parameter values for kinetic rates, Poisson state means, crosstalk probability, and camera exposure period (bin size) are included in the figure captions.

### Data analysis

Given the simulated FRET datasets, the variables inferred included state trajectories, kinetic rates, and FRET efficiencies. In order to generate a probability distribution over each of the variables reported in Figs. 1 and 2 we used Bayesian inference defining the joint probability distribution as proportional to the product of the data’s likelihood and prior distributions resulting in a posterior.

For the likelihood, we built an HMM^8,9^ whose latent variables are the states at a discrete set of time points, with either Poisson or Gaussian emission distributions. Next, weakly informative prior distributions were chosen such that their domains coincide with that of the random variables of interest.

Since posteriors constructed from such a combination of likelihood and priors are analytically intractable, we used Markov Chain Monte Carlo methods^8^ to generate samples. Specifically, we adopted a Gibbs algorithm^8^ where variables of interest—the state trajectory, kinetic rates, and FRET efficiencies, were iteratively sampled in a sequential manner. Details can be found in our code.

## Data Availability

Our simulated datasets are available online^15^. Any other relevant data is available from the corresponding author on request.

## Code availability

Our simulated dataset generation code and HMM analysis is available online^15^. Any other relevant code is available from the corresponding author on request.
